# Wernicke Encephalopathy in Critical Illness: A Diagnostic Challenge With Evolving MRI Findings

**DOI:** 10.7759/cureus.117928

**Published:** 2026-10-14

**Authors:** Tabitha C Mangao, Remmel John R Mangao, Amirhossein H Zangiabadi

**Affiliations:** 1 Neurology, St. George's University School of Medicine, Los Angeles, USA; 2 Stroke and Neurology, St. Francis Medical Center, Lynwood, USA

**Keywords:** critical illness patients, intravenous thiamine, serial mri, wernicke’s, wernicke’s encephalopathy (we)

## Abstract

Wernicke encephalopathy (WE) is an acute neurologic disorder caused by thiamine deficiency that can result in significant neurologic morbidity if not promptly recognized and treated. Diagnosis may be particularly challenging in critically ill patients, in whom altered mental status may be attributed to other metabolic, infectious, or neurologic processes. Magnetic resonance imaging (MRI) can support the diagnosis, although characteristic abnormalities are not always present.

We present a 43-year-old woman with approximately 2 months of poor oral intake who developed progressive encephalopathy during hospitalization for multiple acute medical conditions. Initial brain MRI showed no characteristic findings of WE. Despite treatment of her underlying illnesses and correction of several metabolic abnormalities, her mental status continued to deteriorate. On hospital day eight, she experienced a cardiac arrest with return of spontaneous circulation after approximately two minutes. Repeat MRI later that day demonstrated new symmetric T2-weighted fluid-attenuated inversion recovery (T2/FLAIR) abnormalities involving the mammillary bodies, dorsomedial thalami, tectal plate, periaqueductal gray matter, region surrounding the third ventricle, and posterior frontal cortex, suggestive of WE. Collateral history subsequently revealed heavy alcohol use, and high-dose intravenous thiamine was initiated. Her neurologic examination improved over the following days, while serial MRI on hospital days 16 and 27 demonstrated marked improvement and near-resolution of the characteristic abnormalities, respectively. Although neurologic improvement followed thiamine administration, concurrent recovery from cardiac arrest and systemic illness limits attribution of this improvement to thiamine alone.

This case demonstrates the diagnostic challenge of WE in the setting of critical illness and competing causes of encephalopathy. An initially nondiagnostic MRI does not rule out WE, and continued clinical suspicion with prompt empiric thiamine administration remains important when the diagnosis is suspected.

## Introduction

Wernicke encephalopathy (WE) is an acute neurologic disorder caused by thiamine deficiency and is commonly associated with chronic alcohol use and malnutrition [1,2]. It is classically characterized by the triad of altered mental status, ophthalmoplegia, and ataxia; however, the complete triad is uncommon, making clinical recognition challenging [1,2]. If left untreated, WE can result in persistent neurologic impairment and potentially death, making early recognition and thiamine administration essential [3].

Primary care practitioners and other clinicians involved in the initial evaluation of acutely ill patients may encounter WE among patients presenting with altered mental status, particularly when multiple potential causes of encephalopathy are present. Brain imaging can help evaluate alternative causes of encephalopathy and support the diagnosis of WE. Magnetic resonance imaging (MRI) can demonstrate characteristic abnormalities involving the medial thalami, mammillary bodies, periaqueductal gray matter, and regions surrounding the third ventricle [4]. However, characteristic findings are not universally present, and an initially nondiagnostic MRI does not exclude WE [4]. Here, we present a patient with progressive encephalopathy in the setting of critical illness whose initial brain MRI was nondiagnostic, followed several days later by the development of MRI abnormalities concerning for WE. Serial imaging subsequently demonstrated marked regression of these abnormalities following high-dose intravenous thiamine. This case highlights the diagnostic challenge of recognizing WE amid competing causes of encephalopathy and the importance of continued clinical suspicion when initial evaluation is unrevealing. 

## Case presentation

A 43-year-old woman with hemoglobin E thalassemia and chronic anemia presented to the emergency department (ED) with approximately two months of progressive generalized weakness, poor appetite, and markedly decreased oral intake. Her weakness had progressed to the point that she had difficulty getting out of bed. She additionally reported diffuse pain greater than her baseline, intermittent vomiting, dysuria, subjective fevers, and recent discoloration of her skin. On presentation, she was alert and oriented but appeared pale and jaundiced, with right-sided abdominal tenderness. She weighed 45 kg at a height of 160 cm, corresponding to a body mass index of 17.57 kg/m². Initial laboratory evaluation was notable for severe anemia, hypokalemia, metabolic acidosis, acute kidney injury, hypoalbuminemia, and elevated lactate. She received two units of packed red blood cells in the ED. Subsequent evaluation demonstrated a urinary tract infection and indirect hyperbilirubinemia concerning for hemolysis. Computed tomography (CT) of the abdomen demonstrated mild fat stranding surrounding the pancreatic tail, and lipase was 119 U/L, consistent with mild acute-on-chronic pancreatitis. Imaging also demonstrated cholelithiasis. Her early hospital course was additionally complicated by hypomagnesemia and severe hypophosphatemia requiring intravenous electrolyte replacement.

Alterations in mental status became apparent early in the hospitalization. By hospital day (HD) 1, she was described as confused, and on HD2, she was increasingly altered and difficult to arouse. Brain MRI obtained on HD2 demonstrated no acute infarction, hemorrhage, mass, or other acute intracranial abnormality. Scattered periventricular hyperintensities were present on T2-weighted/fluid-attenuated inversion recovery (T2/FLAIR) and considered nonspecific, with no suggestive findings of WE. Over the following several days, she remained lethargic and intermittently somnolent despite continued treatment of her medical and metabolic abnormalities.

On HD5, the patient developed transient stupor, aphasia, right facial weakness, and right-sided weakness, prompting neurologic evaluation for possible seizure activity. Initial examination documented decreased attention and sensation on the right, with limited movement of the right extremities. During neurologic evaluation, she had her eyes closed but followed commands, resisted eye opening, and developed upper-extremity shaking when her eyes were opened; she was subsequently able to lift all four extremities against gravity. Routine 60-minute electroencephalography (EEG) demonstrated mild cortical cerebral dysfunction consistent with possible toxic-metabolic encephalopathy, without seizure activity. A CT head obtained the following day was also negative for acute pathology. Neurology did not identify evidence of epileptic seizure, and antiseizure medication was not initiated.

Early on the morning of HD8, the patient developed sinus bradycardia with a heart rate in the 30s and was subsequently found unresponsive and pulseless. The rhythm progressed to sinus arrhythmia followed by asystolic cardiac arrest. Cardiopulmonary resuscitation was initiated, and return of spontaneous circulation was achieved after approximately two minutes of resuscitation and one dose of epinephrine. She was subsequently intubated and transferred to the intensive care unit for post-cardiac arrest care and management of acute hypoxic/hypercapnic respiratory failure. The etiology of the cardiac arrest remained uncertain, with sepsis, metabolic derangements, severe anemia, and an acute respiratory event considered possible contributors. Transthoracic echocardiography performed the day before the arrest demonstrated preserved left ventricular systolic function. Post-arrest laboratory testing showed no severe hypokalemia or hypomagnesemia, improved hemoglobin following prior transfusion, and an elevated lactate. Immediately following the arrest, her neurologic examination was poor, with coma, reactive pupils, absent gag and oculocephalic reflexes, and no withdrawal of the extremities to painful stimuli. Post-arrest repeat EEG demonstrated markedly reduced cerebral activity consistent with diffuse structural injury or severe toxic-metabolic encephalopathy, without seizure activity.

A repeat brain MRI obtained at 4:30 PM on HD8, approximately 11-12 hours after the cardiac arrest, demonstrated new symmetric T2/FLAIR hyperintensities involving the mammillary bodies, dorsomedial thalami, tectal plate, periaqueductal gray matter, periventricular region surrounding the third ventricle, and cerebral cortex predominantly involving the posterior frontal high convexities. No corresponding diffusion-weighted imaging (DWI) abnormalities were reported, and there was no evidence of acute hemorrhage, mass, or midline shift. The distribution of the deep gray and periventricular abnormalities was concerning for WE, while the posterior frontal cortical abnormalities could also reflect hypoxic-ischemic injury following cardiac arrest. Collateral history obtained from the patient's husband following the arrest revealed heavy alcohol use. A serum thiamine level was not obtained. Following the MRI findings and collateral history, high-dose intravenous thiamine was initiated, with the first 500 mg dose administered the evening of HD8.

The patient subsequently demonstrated improvement in her neurologic examination. By HD9, she had regained cough and gag reflexes, opened her eyes, moved her head spontaneously, and withdrew all four extremities to painful stimulation. Attempts to decrease sedation resulted in agitation and spontaneous movement. She continued to receive 500 mg of intravenous thiamine every eight hours. By HD10, she opened her eyes, visually tracked, and followed simple commands by nodding yes or no, allowing successful extubation. On HD11, the thiamine regimen was transitioned to 200 mg intravenously every eight hours, which was continued through HD31. Despite improvement from her immediate post-arrest examination, her neurologic recovery remained incomplete, with persistent lethargy, limited verbal output, generalized weakness, and dysphagia.

Serial neuroimaging demonstrated substantial interval improvement after initiation of thiamine therapy. Repeat brain MRI on HD16 demonstrated a marked interval decrease in T2/FLAIR signal abnormalities involving the mammillary bodies, dorsomedial thalami, tectal plate, periaqueductal gray matter, and periventricular region surrounding the third ventricle, with resolution of the previously observed posterior frontal cortical signal abnormalities. A subsequent MRI on HD27 demonstrated near-resolution of the abnormalities originally identified on HD8. Signal abnormalities surrounding the third ventricle and within the periaqueductal region had essentially resolved, with only minimal residual signal abnormalities involving the posterior thalami and posterior frontal cortex and no new intracranial abnormalities (Figure 1). 

**Figure 1:**
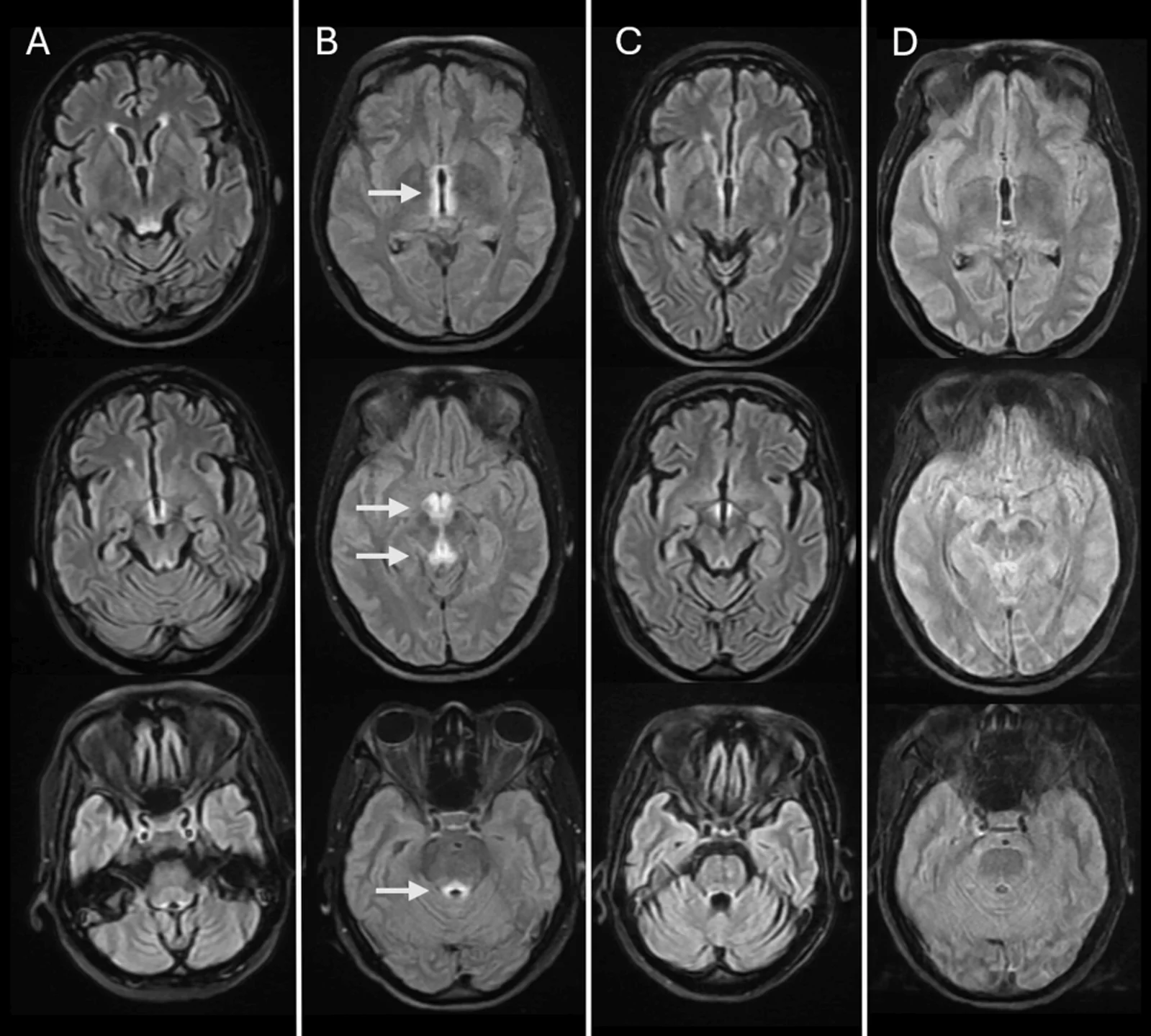
Serial axial T2-weighted/fluid-attenuated inversion recovery (T2/FLAIR) magnetic resonance imaging (MRI) demonstrating evolving findings of Wernicke encephalopathy (WE) (A) Initial MRI obtained on hospital day (HD) 2 demonstrates no characteristic Wernicke-associated signal abnormalities. (B) Repeat MRI on HD8 demonstrates new symmetric hyperintensities involving (arrows from top to bottom) characteristic periventricular region surrounding the third ventricle, the mamillary bodies, tectal plate/colliculi, and periaqueductal grey matter. (C) Follow-up MRI on HD16 demonstrates marked interval reduction in the previously observed signal abnormalities after initiation of high-dose intravenous thiamine. (D) MRI obtained on HD27 demonstrates near-resolution of the previously identified abnormalities.

Her remaining hospitalization was complicated by persistent dysphagia requiring percutaneous endoscopic gastrostomy placement, profound generalized weakness, recurrent hypoxic respiratory failure, aspiration pneumonia, and subsequent reintubation and tracheostomy. At the time of discharge, she remained ventilator-dependent and profoundly debilitated. She was awake and nonverbal, made brief eye contact, and could respond to requests with blinking or a small smile, although she was unable to move her extremities on the documented discharge examination. She was discharged to a long-term acute care facility for continued management and rehabilitation.

## Discussion

Wernicke encephalopathy is an acute neurologic disorder resulting from thiamine deficiency; it remains challenging to diagnose because its clinical manifestations vary. Although classically characterized by the triad of altered mental status, ophthalmoplegia, and ataxia, the complete triad is present in only a minority of patients, limiting its utility as a diagnostic requirement [1,2]. Caine et al. proposed broader clinical criteria requiring two of four findings: dietary deficiency, oculomotor abnormalities, cerebellar dysfunction, or altered mental status/mild memory impairment. These criteria emphasize the importance of recognizing WE even in the absence of its classic presentation. In this patient, prolonged poor oral intake and progressive altered mental status were documented, fulfilling two of the clinical criteria proposed by Caine et al. [1].

This patient presented a particular diagnostic challenge because progressive encephalopathy developed in the setting of several competing explanations for altered mental status. Severe anemia, urinary tract infection, pancreatitis, acute kidney injury, and profound electrolyte abnormalities were identified early in her hospitalization, while a subsequent seizure-like episode raised an additional neurologic consideration. Furthermore, the cardiac arrest on HD8 introduced the possibility of hypoxic-ischemic injury as an explanation for her subsequent severe neurologic impairment. Importantly, however, altered mental status preceded the arrest by several days and continued to progress despite treatment of her underlying medical conditions. Before the cardiac arrest, her previously severe electrolyte abnormalities had improved, with potassium, magnesium, and phosphorus returning to near-normal levels, although mild hyponatremia, hypoalbuminemia, and anemia persisted despite prior transfusion. In retrospect, she also had substantial risk factors for thiamine deficiency, including approximately two months of markedly reduced oral intake, low body mass index, and later-reported heavy alcohol use. Although alcohol use is a well-recognized risk factor for WE [2], thiamine deficiency may also occur during critical illness because of inadequate nutritional intake and increased metabolic requirements [5]. In a prospective study of patients with sepsis, Donnino et al. found that some critically ill patients presented with thiamine deficiency while others developed deficiency during the first 72 hours of illness [5]. Thus, critical illness may compound preexisting nutritional vulnerability and further obscure recognition of WE.

Neuroimaging was particularly informative in this case because of the evolution observed across serial MRI examinations. The initial MRI on HD2 showed no characteristic findings of WE, whereas repeat imaging on HD8 demonstrated symmetric T2/FLAIR abnormalities involving the mammillary bodies, dorsomedial thalami, tectal plate, periaqueductal gray matter, periventricular region surrounding the third ventricle, and posterior frontal cortex. These locations are consistent with the characteristic distribution described in WE [2,4]. However, MRI is supportive rather than exclusionary in the diagnosis of WE. Antunez et al. reported a sensitivity of only 53% and specificity of 93% for MRI in diagnosing acute WE, demonstrating that the absence of characteristic abnormalities does not reliably exclude the disorder [6]. More recently, Silva et al. found suggestive T2/FLAIR abnormalities in approximately one-third of clinically diagnosed patients who underwent MRI. They also found that the odds of demonstrating typical WE imaging findings decreased by 5.3% for each additional 100 mg of thiamine administered before imaging (p=0.046) [7]. Prior thiamine exposure may reduce the likelihood of detecting typical MRI abnormalities; however, this association is based on observational data and should be interpreted cautiously. The initially unrevealing MRI in this patient therefore illustrates the importance of maintaining clinical suspicion when neurologic deterioration continues despite nondiagnostic early imaging.

The subsequent evolution of her imaging provides additional support for the diagnosis. On HD16, repeat MRI demonstrated marked reduction in the previously observed abnormalities. By HD27, 19 days after treatment initiation, the abnormalities had nearly resolved. Follow-up imaging studies have similarly demonstrated regression of WE-associated abnormalities after thiamine administration. Zhong et al. described characteristic periaqueductal, periventricular, and medial thalamic abnormalities in nonalcoholic WE and emphasized the utility of MRI not only in diagnosis but also in evaluating disease evolution and prognosis [8]. The sequence in this patient-from initially nondiagnostic imaging to characteristic abnormalities during clinical deterioration, followed by marked regression after treatment-provides an illustrative radiographic course.

Treatment of suspected WE should be initiated promptly because delayed thiamine replacement may result in irreversible neurologic injury. Although dosing recommendations vary, parenteral thiamine is preferred when WE is suspected, and high-dose regimens are recommended by several expert guidelines and reviews [3,9]. In this case, no serum thiamine level was obtained; however, laboratory confirmation should not delay treatment when WE is clinically suspected [10]. Following recognition of the characteristic MRI abnormalities and collateral history of heavy alcohol use, the patient received 500 mg of intravenous thiamine every eight hours beginning on HD8. Within the following days, her examination improved from coma with absent brainstem reflexes and no withdrawal to eye opening, visual tracking, withdrawal of all extremities, and simple command following, permitting extubation on HD10. This temporal relationship between thiamine administration and subsequent clinical and radiographic improvement further supported the diagnosis of WE. However, concurrent recovery from cardiac arrest and treatment of her other systemic abnormalities preclude attributing the neurologic improvement solely to thiamine administration.

Despite near-resolution of the characteristic MRI abnormalities, the patient's functional recovery remained incomplete. Her prolonged hospitalization was complicated by recurrent respiratory failure, aspiration pneumonia, severe generalized weakness, dysphagia, reintubation, and eventual tracheostomy. At discharge, she remained profoundly debilitated and required transfer to a long-term acute care facility. This discordance between substantial radiographic improvement and persistent clinical disability underscores the difficulty of assessing neurologic outcome in WE occurring alongside prolonged critical illness. Her residual deficits cannot be attributed exclusively to thiamine deficiency, particularly given the multiple potential contributors to neurologic injury and deconditioning.

This case highlights the importance of maintaining suspicion for WE in critically ill patients with nutritional risk factors and progressive unexplained encephalopathy, even when more immediate metabolic, infectious, or post-arrest explanations are present. An initially nondiagnostic MRI should not exclude WE when the clinical course remains concerning, and repeat imaging may reveal characteristic abnormalities as the disease evolves. Because treatment is time-sensitive and laboratory confirmation is not required before therapy, empiric parenteral thiamine should be initiated promptly when WE is suspected. The Royal College of Physicians recommends intravenous thiamine 500 mg three times daily for 2-3 days, followed by 250 mg intravenously once daily for 5 days and then oral thiamine supplementation [11]. Serial imaging in this case additionally demonstrated marked regression of characteristic abnormalities following treatment, emphasizing both the dynamic radiographic nature of WE and the potential reversibility of WE-associated MRI abnormalities.

## Conclusions

Wernicke encephalopathy should remain a diagnostic consideration in patients with nutritional risk factors and progressive unexplained encephalopathy. This is particularly important when critical illness obscures the cause of neurologic deterioration. An initially nondiagnostic MRI does not exclude Wernicke encephalopathy, and repeat imaging may demonstrate characteristic abnormalities as the disease evolves. Because delayed treatment can result in irreversible neurologic injury, thiamine supplementation should not be postponed while awaiting laboratory or radiographic confirmation. This case illustrates the evolving radiographic course of Wernicke encephalopathy and emphasizes the importance of continued clinical suspicion and prompt treatment.
